# Formal Modelling and Runtime Verification of Autonomous Grasping for Active Debris Removal

**DOI:** 10.3389/frobt.2021.639282

**Published:** 2022-01-27

**Authors:** Marie Farrell, Nikos Mavrakis, Angelo Ferrando, Clare Dixon, Yang Gao

**Affiliations:** ^1^ Department of Computer Science, Maynooth University, Maynooth, Ireland; ^2^ Department of Electronics, University of York, York, United Kingdom; ^3^ Department of Computer Science, University of Genova, Genova, Italy; ^4^ Department of Computer Science, University of Manchester, Manchester, United Kingdom; ^5^ STAR-Lab, Surrey Space Centre, University of Surrey, Guildford, United Kingdom

**Keywords:** autonomous grasping, formal verification, requirements elicitation, runtime verification, formal methods, active debris removal

## Abstract

Active debris removal in space has become a necessary activity to maintain and facilitate orbital operations. Current approaches tend to adopt autonomous robotic systems which are often furnished with a robotic arm to safely capture debris by identifying a suitable grasping point. These systems are controlled by mission-critical software, where a software failure can lead to mission failure which is difficult to recover from since the robotic systems are not easily accessible to humans. Therefore, verifying that these autonomous robotic systems function correctly is crucial. Formal verification methods enable us to analyse the software that is controlling these systems and to provide a proof of correctness that the software obeys its requirements. However, robotic systems tend not to be developed with verification in mind from the outset, which can often complicate the verification of the final algorithms and systems. In this paper, we describe the process that we used to verify a pre-existing system for autonomous grasping which is to be used for active debris removal in space. In particular, we formalise the requirements for this system using the Formal Requirements Elicitation Tool (FRET). We formally model specific software components of the system and formally verify that they adhere to their corresponding requirements using the Dafny program verifier. From the original FRET requirements, we synthesise runtime monitors using ROSMonitoring and show how these can provide runtime assurances for the system. We also describe our experimentation and analysis of the testbed and the associated simulation. We provide a detailed discussion of our approach and describe how the modularity of this particular autonomous system simplified the usually complex task of verifying a system post-development.

## 1 Introduction

Removing orbital debris is an important activity to maintain easy access to space and uninterrupted orbital operations. Approximately 18% of catalogued debris consists of launch products such as spent rocket stages [ESA (online)]. Active Debris Removal is the field of studying methods for removing such debris from orbit, and a variety of methods have been proposed for capturing and removal (Shan et al., 2016). Current approaches to removing these items include the use of autonomous robotics which are equipped with an arm to capture this kind of debris (Mavrakis and Gao, 2019). A central part of the process of removing space debris is in identifying a suitable grasping point on the target surface and ensuring a stable grasp.

Verifying that autonomous space robotic software behaves correctly is crucial, particularly since such software tends to be mission-critical where a software failure can lead to mission failure. Formal verification is a technique that is used to reason about the correctness of a software system with the output providing a proof of correctness that the software behaves correctly, according to the identified requirements (Luckcuck et al., 2019). Robotic systems tend not to be developed with verification in mind from the outset, which often makes the verification of the final algorithms and systems more difficult. In this paper, we describe the process that we used to verify a pre-existing system for autonomous grasping which is to be used for active debris removal in space. In this work, the modularity of the system was particularly beneficial when defining requirements that could subsequently be formally modelled, verified and monitored and monitored.

In this paper, we report on our experience of using an existing formal method, the Dafny program verifier (Leino, 2013), to formally verify our previously developed algorithm for grasping a spacecraft motor nozzle (Mavrakis and Gao, 2019). This takes the usual approach of verifying a pre-existing algorithm and an initial effort was originally presented in (Farrell et al., 2020). However, we augment this process by returning to requirements elicitation, using FRET, which would ideally be done prior to implementation, to successfully define useful requirements for verification purposes. This work extends our original paper by incorporating runtime monitors and detailed experiments. We have also refactored and expanded the original Dafny model in light of the FRET requirements that we produce in this paper. We emphasise that the scope of this paper is not to present the grasp planning algorithm itself in great detail, but rather to discuss our approach to verifying this pre-existing algorithm.

This work demonstrates that, although requirements are ideally formally defined from the outset, there is value in employing these techniques later in the project to identify the essential properties for verification and thus to streamline the process of verifying a pre-existing algorithm. For requirements elicitation, we use the Formal Requirements Elicitation Tool (FRET), as developed by NASA (Giannakopoulou et al., 2020). We also build an AADL model of the system to explicitly identify its components, which provided a useful point of reference when articulating the requirements in FRET (Feiler et al., 2006). Further, we demonstrate how the artefacts of our verification-focused development process can be used to generate runtime monitors for the system and we use the ROSMonitoring tool^1^ for runtime verification. We evaluate our runtime monitors via fault injection and, experimentation on the testbed and the associated simulation of the system.

This paper is structured as follows. Section 2 describes the relevant background material relating to the formal verification of space systems. We include an AADL model of the system in Section 3, describe the corresponding requirements and their encoding in FRET. We use the Dafny program verifier to verify the requirements relating to particular software components in Section 4. Then, in Section 5, we present the runtime monitors for the system and show how they are used to verify a subset of the system requirements. Section 6 describes our experimental evaluation of these runtime monitors using fault injection. We reflect on our approach in Section 7 and Section 8 concludes.

## 2 Related Work

Increased autonomy is particularly desirable in the space industry to support space missions since remote operation may be problematic or impossible due to distance, time lags or communication issues and manual operation may be hazardous or impossible. In fact, autonomy can save time and prevent failures by removing the need for human intervention. Many space systems provide important services and so it is vital that they are correctly and robustly verified. We discuss a number of approaches related to the verification of space systems in this section.

A comprehensive overview of the state-of-the-art verification and validation for autonomous space robotics can be found in (Cardoso et al., 2021). This includes both formal (model checking, theorem proving and runtime verification) and non-formal techniques (testing, simulation). Related, a recent survey of formal specification and verification techniques for autonomous robotic systems has revealed that, although there are many tools and techniques available, improvements are still required for their successful deployment in large, complex and autonomous systems (Farrell et al., 2018; Luckcuck et al., 2019). Given modular robot architectures composed of distinct subsystems, different types of verification can potentially be used for different components, as described in (Farrell et al., 2019b; Cardoso et al., 2020a; Cardoso et al., 2020b), as some verification techniques may be more appropriate than others for certain subsystems. Our work also uses multiple distinct verification techniques (Dafny and ROSMonitoring) to verify different components/aspects of the system. Additionally, first-order logic can be used to specify the assumptions on inputs and guarantees on outputs for each subsystem so that we can ensure that the system architecture satisfies these.

A related assume-guarantee framework has been proposed for the verification of autonomous space systems (Brat et al., 2006). They target systems that are composed of 3 layers (planning, execution and functional layers) and they make use of model-checking, static analysis, synthesis and testing to demonstrate their approach. In other work, the authors compared tools for static analysis, model-checking and runtime verification against traditional testing of rover flight software (Brat et al., 2004). Each of these formal techniques outperformed testing when locating concurrency errors. Other rover verification research includes (Bourbouh et al., 2021) which uses FRET, CoCoSpec and Event-B to verify an autonomous rover use case. The verification artefacts are subsequently collected and used to form an assurance case. This work focuses primarily on static verification whereas here we also include runtime monitors for more dynamic properties.

Webster et al. (2020) propose using a combination of different types of verification (both formal and non-formal) to improve the confidence in the overall system. They apply formal verification via model checking, simulation based testing and experiments with the physical robot to a collaborative manufacturing scenario, relating to a handover task. The outcomes from the different verification methods are used to inform, improve and update the inputs to the other methods. We have taken a similar approach in this paper where we combine formalised requirements in FRET, theorem proving in Dafny and runtime verification. Section 7 explores the benefits of our approach.

This paper builds on our previous work (Farrell et al., 2020) where we verified part of the grasping algorithm presented in (Mavrakis and Gao, 2019). This paper expands on this by looking at whole-system verification rather than the verification of a specific software component. As a result, we present a refactored Dafny model with more detailed explanations in Section 4. As mentioned in the Introduction, we additionally incorporate runtime monitors and experiments.

In Section 3, we use the Formal Requirements Elicitation Tool which supports translation into CoCoSim for compositional verification (Mavridou et al., 2019). This approach is primarily aimed at Simulink models. Hence, we do not use CoCoSim here because our target is a ROS-based system that has been implemented in Python. Related to this, recent work has used FRET requirements to generate runtime monitors in the Copilot framework which were incorporated into the ICAROUS architecture for autonomous operations of unmanned aircraft. The target language for these monitors was a restricted subset of C, whereas we focus on generating monitors for Python (Dutle et al., 2020).

UML-based formal assertions and runtime monitoring have been employed to verify and validate the flight software for a Brazilian satellite launcher (Alves et al., 2011). The authors collected the data for the runtime monitoring JUnit tests from the associated log files. Related to this, the R2U2 tool (Responsive, Realisable, Unobtrusive Unit) has been used in the development of small satellites (Schumann et al., 2015; Rozier and Schumann, 2016). This work is similar to ours, however they do not formally elicit requirements in the way that we have and we use a theorem proving rather than a model checking approach for static verification.

Event-B specifications have been combined with probabilistic properties to derive reconfigurable architectures for an on-board satellite system (Tarasyuk et al., 2012). This work uses PRISM to check for both the derivation (via formal refinement) of the system from its specification and the probabilistic assessment of their reliability and performance. Our approach uses formalised requirements to derive the properties that are to be verified which promotes traceability of requirements.

This work uses Dafny which was chosen because it closely resembles Python so it was relatively straightforward to translate the autonomous grasping algorithm into Dafny. There are a multitude of other formal methods that we could have chosen instead of Dafny, including Spec# (Barnett et al., 2004), Spark Ada (Carré and Garnsworthy, 1990), Frama-C (Cuoq et al., 2012), etc. However, we found that Dafny was sufficiently expressive for the properties that we wanted to verify and, in related work (Farrell et al., 2019a, 2020), we have found it accessible when working with other researchers that are not familiar with formal methods. This second point is important because the authors of this paper are a combination of formal methods researchers and engineers.

## 3 System Overview and Requirements Elicitation

In this section, we provide an overview of the system architecture and describe the associated requirements which were formalised using FRET. From a verification perspective, it is important to first understand the organisation and structure of the system at hand before assigning requirements to specific components. For this step, we employ the widely-used Architecture Analysis and Design Language (AADL).

### 3.1 Architecture Analysis and Design Language Model

**FIGURE 1:**
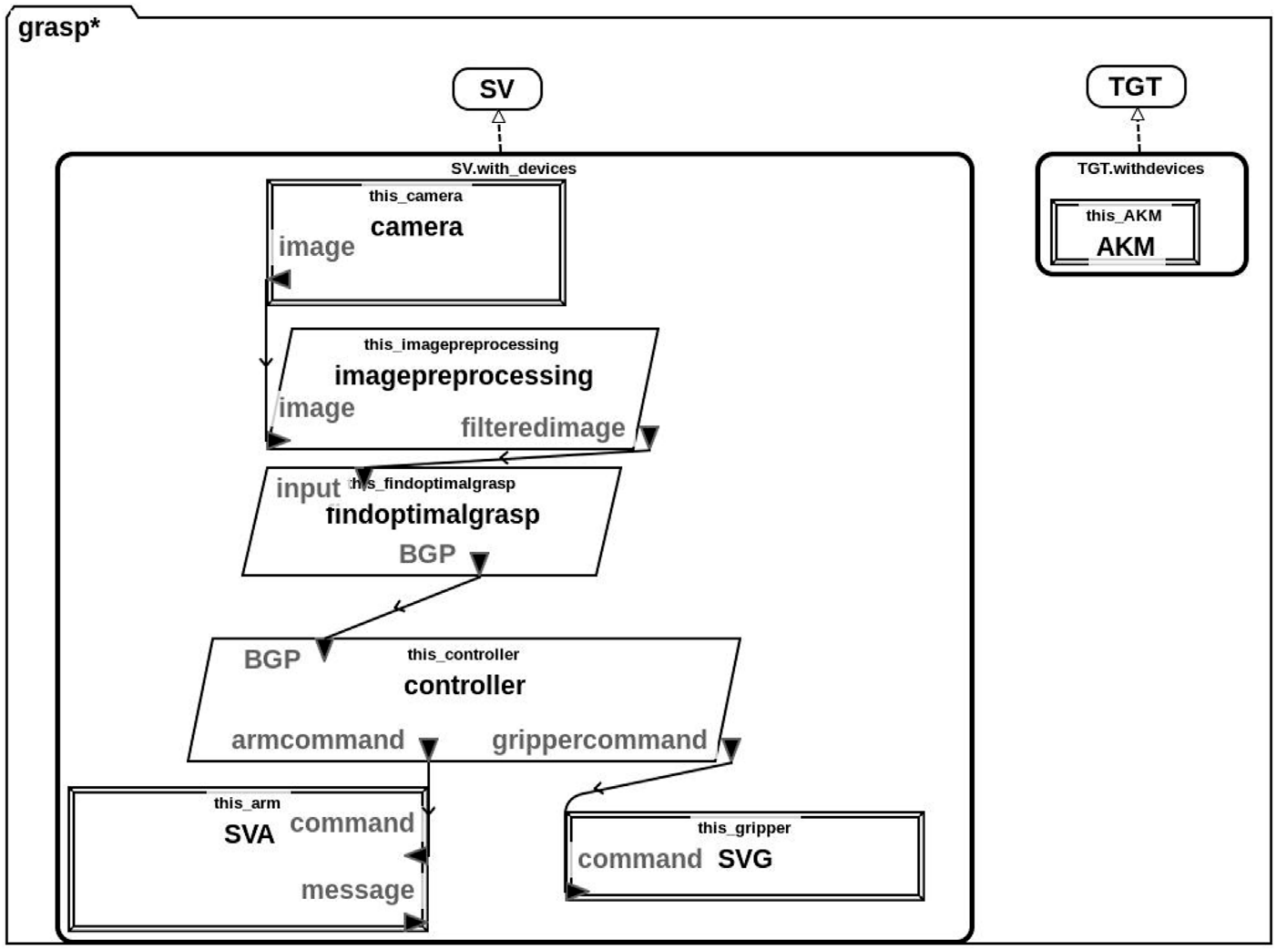
AADL model of the system comprising both the hardware and software components of the service vehicle (SV) and the target (TGT). The SV contains a camera, arm (SVA) and gripper (SVG) as hardware components. Its software components are used to pre-process the input image (imagepreprocessing), calculate the optimal grasp (findoptimalgrasp) and control the arm and gripper (controller). Arrows indicate data flow and variable names are given.

We have used the AADL to devise a model of the system which incorporates both hardware and software components (Feiler et al., 2006). We use this model, as illustrated in Figure 1, as a point of reference when developing the requirements for this system in the next subsection. The task of producing this model allowed us to analyse the system in question and to decompose its functionality. This functional decomposition resulted in some small refactoring of the original Python implementation to facilitate more detailed software verification and to easily include runtime monitors.

In particular, this model contains both the Service Vehicle (SV) and Target (TGT). We also include details about the individual hardware (rectangle) and software (parallelogram) components of these vehicles. Specifically, the SV is equipped with a camera, service vehicle arm (SVA) and service vehicle gripper (SVG) hardware components. Its software components consist of components for image processing, finding the optimal grasp and a hardware controller. The TGT is less sophisticated and its only hardware component is an apogee kick motor (AKM), it does not have any on-board software.

The lines and arrows in Figure 1 represent communication between the various components and we have indicated the data that is to be communicated on each arrow. Specifically, the camera sends an image to the imagepreprocessing component which produces a filteredimage. This filteredimage is sent to the findoptimalgrasp component which returns the BGP (best grasp pose) to the controller. Finally, the controller communicates the appropriate commands to the SVA and SVG.

In our system, we assume a *non-cooperative* target, TGT and this is reflected in the AADL model. Thus, the TGT is not equipped with any software or hardware components other than an apogee kick motor (AKM) shown in this diagram.

### 3.2 Requirements Elicitation

We use the AADL model that was outlined above (Figure 1) as a point of reference for defining both system-level and component-level requirements. In particular, we define the following system-level requirements:

**R1:** *The SV shall grasp the TGT at the BGP and draw it closer.*

**R2:** *The SV shall not collide with the TGT.*

Here, R1 is focused on the correct/intended functionality of the system, whereas, R2 is a safety requirement. From these system-level parent requirements (R1 and R2), we derive the full list of detailed requirements as shown in Table 1. We have given an ID to each requirement in the table (first column) and the second column contains the natural language description of each requirement. The last column contains the FRET formalisation of these requirements (in FRETISH).

**TABLE 1:** Natural language requirements and their corresponding formalisation in FRET based on the components illustrated in our AADL model (Figure 1). We encourage the reader to use this table as a point of reference for specific requirements that are mentioned in the text.

ID	English-Language Description	FRET formalisation
R1	The SV shall grasp the TGT at the BGP and draw it closer.	SV shall satisfy (grasp(TGT, BGP) & closer(SV, TGT))
R1.1	The Camera of the SV shall be positioned at least 0.5 m from the TGT.	Camera shall satisfy distance(Camera, TGT) ≥ 0.5
R1.2	The TGT shall be motionless before contact with the SVA.	TGT shall satisfy if !contact(SVA, TGT) then motionless(TGT)
R1.3	The Camera shall return a valid point cloud.	Camera shall satisfy valid(p)
R1.3.1	The point cloud shall be structured with maximum resolution of 1,280 × 720.	Camera shall satisfy maxRes(p) = 1,280*720
R1.3.2	The point cloud shall not be empty.	Camera shall satisfy length(p) > 0
R1.4	The imagepreprocessing shall return a filtered point cloud.	Imagepreprocessing shall satisfy length(filteredimage) ≤ length(p) & length(filteredimage) > 0
R1.5	findoptimalgrasp shall return the optimal grasp point (BGP) if one exists.	Findoptimalgrasp shall satisfy if exists(BGP) then return(BGP)
R.1.5.1	The BGP shall be optimal according to the criteria: minimum offset from the TGT nozzle edge of 1 cm and finger-surface yaw angle between −20 and 20°.	Findoptimalgrasp shall satisfy offset(BGP, TGT) = 1 & -20 ≤ fingersurfaceyaw & fingersurfaceyaw ≤20
R1.5.2	findoptimalgrasp shall generate several candidate grasping points.	findoptimallgrasp shall satisfy length(grasps) ≥ 0
R1.6	If no BGP exists then findoptimalgrasp shall output an error message.	Findoptimalgrasp shall satisfy if !(exists(BGP)) then printerror
R1.7	Controller shall execute a joint trajectory to reach the BGP.	Controller shall satisfy executeJointTrajectory(SVA, BGP)
R1.8	The SVA shall capture the TGT at the BGP.	SVA shall satisfy captured(TGT) ⇒ contactpoint(SVA, TGT) = BGP
R1.9	The total pulling distance shall be between 0.3 and 0.5 m.	SV shall satisfy totalpullingdistance ≥0.3 & totalpullingdistance ≤0.5
R2	The SV shall not collide with the TGT.	SV shall always satisfy !collide(SV, TGT)
R2.1	The position of the SV shall not be equal to the position of the TGT.	SV shall always satisfy !(position(SV) = position(TGT))
R2.2	The SV shall only make contact with the TGT at the BGP using the SVG.	SV shall always satisfy contactpoint(SVG, TGT) = BGP.
R2.2.1	No part of the SV, other than the SVG shall make contact with the TGT.	SV shall satisfy if !grasped then contactpoint(SV, TGT) = null
R2.2.2	The SVG shall only make contact with the TGT at the BGP (within some margin of error).	SV shall satisfy if grasped then contactpoint(SVG,TGT) = BGP + errormargin
R2.3	The SVG shall apply a force of 180 N once contact has been made with the TGT.	SVG shall satisfy captured(TGT) ⇒ force = 180

The Formal Requirements Elicitation Tool (FRET) (Giannakopoulou et al., 2020) supports the formalisation, understanding and analysis of requirements through a user-friendly interface with intuitive diagrammatic explanations of requirement semantics. Users specify their requirements in restricted natural language, called FRETISH, which embodies a temporal logic semantics.

**FIGURE 2:**
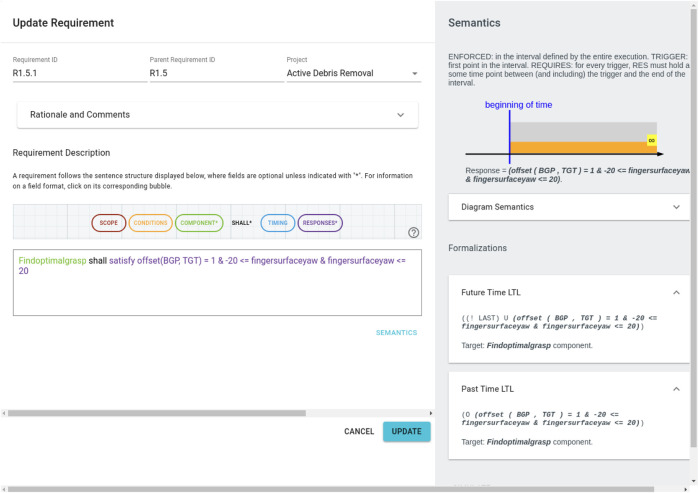
Screenshot from FRET corresponding to R1.5.2 which describes how the best grasp pose (BGP) is chosen. Each FRET requirement is given an ID (top left), there is an option to include Rationale and Comments which can be useful to promote traceability. Requirements are described in FRETISH with automatic syntax highlighting. Then, FRET generates both diagrammatic and LTL semantics (right hand side) corresponding to the requirment.

As an example, we include a screenshot from the FRET tool in Figure 2 corresponding to R1.5.2 from Table 1. Here, we have defined a parent-child relationship between R1.5 and R1.5.2 which allows us to maintain the hierarchy of requirements as indicated by the IDs in Table 1. On the right hand side, the FRET tool displays a formal semantics for this requirement in both future time and past time linear temporal logic (LTL). For users who are not familiar with this semantics/notation, FRET also includes a diagrammatic representation of what this requirement means. Users can enter natural-language descriptions in the “Rationale and Comments” section shown in Figure 1.

FRET is particularly useful as an intermediate step between the development of natural language requirements and the formalisation of these requirements in a formal verification tool. This is because FRET provides a template:

SCOPE CONDITION COMPONENT SHALL TIMING RESPONSES

which enforces a logical structure relating the pre-conditions for a specific component (on the left of SHALL) to the associated post-conditions (on the right of SHALL). Although not all formal methods support the temporal semantics used by FRET, FRET requirements can still be useful and more straightforward to formalise than natural language requirements. Note that the FRETISH editor dynamically colours the entered text corresponding to the designated fields in “Requirement Description”.

In the above template, the COMPONENT field is mandatory to indicate which system component a specific requirement applies to. The SHALL keyword is also mandatory and specifies that the identified component *must* conform to the requirement. The last mandatory field is RESPONSE which is currently of the form satisfy R where R is a non-temporal boolean valued formula.

With respect to the optional fields, SCOPE indicates that a requirement is only relevant for particular scopes of the system behaviour. CONDITION describes a point after which the requirement must hold and TIMING defines the point at which the response should occur.

Although FRET can export CoCoSpec verification code (Mavridou et al., 2019), this exported code was not used for this case study since it required Simulink-based tools and our system was implemented using ROS in Python. Instead, we used the FRETISH representation of the requirement and the associated LTL semantics that is generated by FRET to guide our Dafny verification and runtime monitor generation.

In the subsequent sections, we describe how we verify these requirements.

## 4 Formal Modelling and Verification with Dafny

The work presented in this section was originally described in (Farrell et al., 2020). However, this section provides more detailed models and discussion in relation to the requirements identified in Table 1. In our prior work (Farrell et al., 2020), we focused our Dafny verification effort on three key requirements. These correspond to R1.5, R1.5.1, R1.5.2 and R1.6 as listed in Table 1. Our Dafny model was primarily concerned with functional correctness to demonstrate that the algorithm for choosing the BGP behaves correctly. We have modified the Dafny model from (Farrell et al., 2020), so that it more accurately reflects the architecture presented in the AADL model in Figure 1 and provided more detailed implementations of the helper functions.

Dafny is a formal verification system that is used in the static verification of functional program correctness. Users provide specification constructs e.g. pre-/post-conditions, loop invariants and variants (Leino, 2010). Programs are translated into the Boogie intermediate verification language (Barnett et al., 2005) and then the Z3 automated theorem prover discharges the associated proof obligations (De Moura and Bjørner, 2008).

**FIGURE 3:**
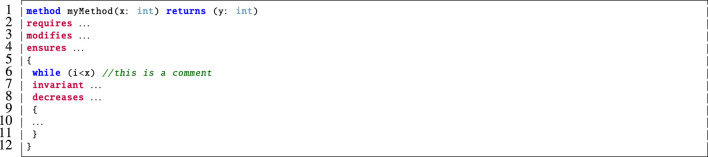
The basic structure of a method with specification constructs in Dafny.

The basic structure of a method in Dafny is outlined in Figure 3. Here, the **requires** keyword on line 2 is used to indicate the pre-condition for the method, the **modifies** keyword on line 3 specifies which of the input variables the method is allowed to modify and the **ensures** keyword on line 4 accounts for the method’s post-condition. The user specifies the loop **invariant** on line 7 which is used by the underlying SMT solver to reason about the correctness of the loop and to prove that the post-condition is preserved and the **decreases** clause on line 8 corresponds to a loop variant for proving loop termination.

We devised a formal model of our grasp planning algorithm in Dafny including the definition of two particular methods. One which captures the functionality of Algorithm 1 and another which specifically focuses on how the optimal grasp is selected. We also had to model a series of helper functions since the Python implementation of the grasping algorithm uses library functions which are not available in Dafny. For the purposes of verification, we specify pre-/post-conditions for each of these methods. We specifically focus on verifying that the chosen grasp is valid, correct, and optimal with respect to the defined criteria (Mavrakis and Gao, 2019). We also verify that the helper functions behave as expected, as well as the usual suite of standard program correctness properties (e.g. loop termination, etc.). To accurately correspond to Figure 1, we also refactored our Dafny model and created a separate method corresponding to the imagepreprocessing component shown in Figure 1. This separation was not present in our original model (Farrell et al., 2020).

### 4.1 Overview: Autonomous Grasping Algorithm

This paper focuses on an autonomous grasping use case where a robotic arm shall grasp a piece of space debris. The algorithm which captures the autonomous behaviour of the system (Mavrakis and Gao, 2019), implemented in Python, extracts a point cloud of the nozzle removing outlier points (via depth removal, downsampling and filtering) and calculates the Zero Moment Shift (ZMS) of every point, which describes the surface smoothness. A 4D feature vector is formed for every point, including its 3D coordinates and its ZMS norm. The vectors are fed to a clustering algorithm that extracts graspable patches, and Principal Component Analysis (PCA) is applied on each patch to extract a 3D coordinate frame that constitutes a grasping pose (finger positions on the nozzle surface). The best grasping pose is selected according to pre-defined reachability criteria.

**FIGURE 4:**
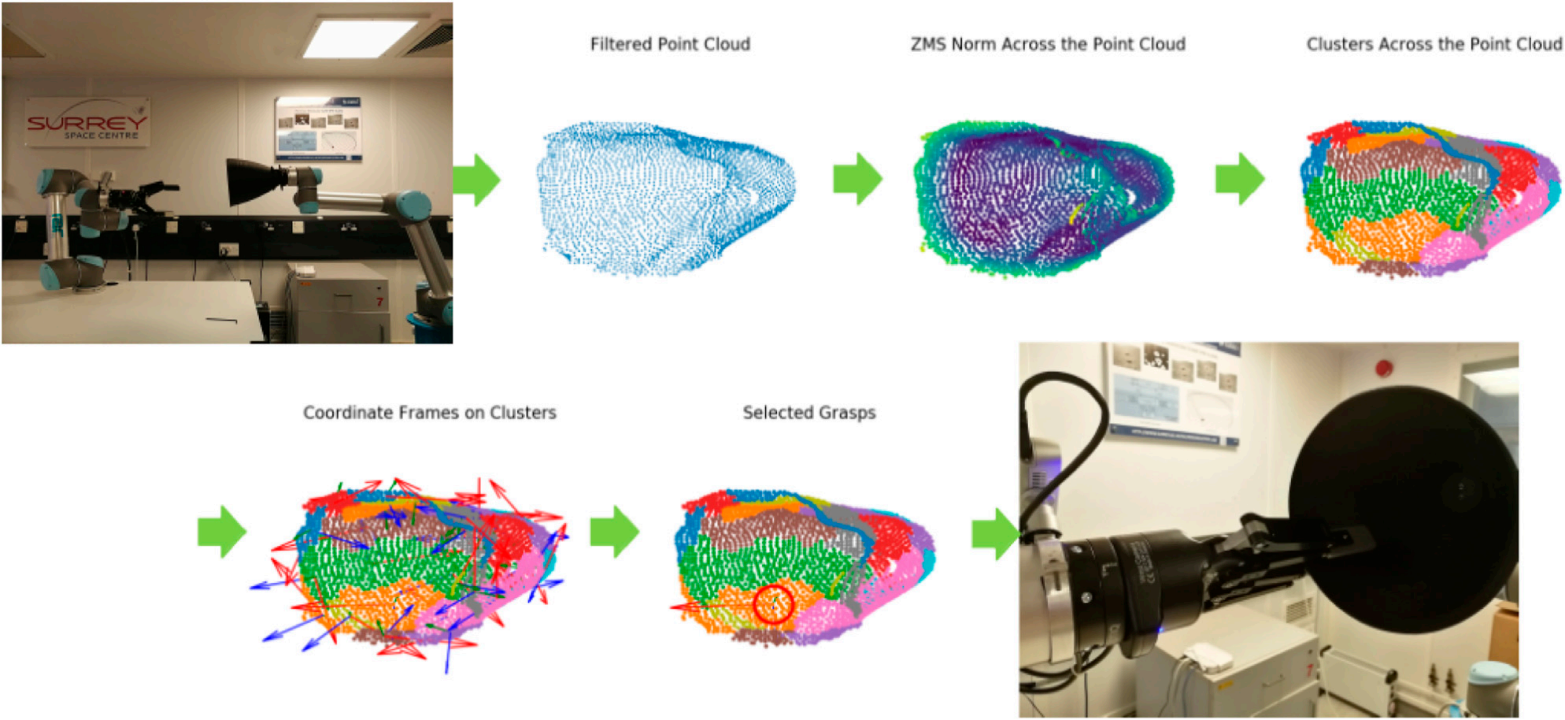
Execution of the grasping algorithm on a robotic testbed, for a 3D printed nozzle, as presented by Mavrakis and Gao (2019). The intermediate steps for point cloud processing and grasp synthesis are shown.

The grasping algorithm to be verified has been presented analytically by Mavrakis and Gao (2019). In this paper, we briefly describe the grasp synthesis process. The reader is encouraged to study the original paper for additional information about the detailed functionality of the grasping algorithm that we verify in this paper. The algorithm has been developed for the capturing of spacecraft engine nozzles, be it spent rocket stages or satellites. The unique curvature of nozzles, their presence in most spacecraft and rocket bodies, as well as their structural robustness make them ideal contact points for capturing of both cooperative satellites and uncooperative space debris. An execution of the algorithm steps is shown in Figure 4.

In order to verify the algorithm, we first translate these intermediate steps into pseudocode description. The pseudocode decomposes the functionality of the algorithm into modules with pre-defined inputs and outputs. This helps us to design pre- and post-conditions both for each module and the algorithm as a whole, enabling the verification of numerous properties. The pseudocode is given in Algorithm 1.

**Figure:** 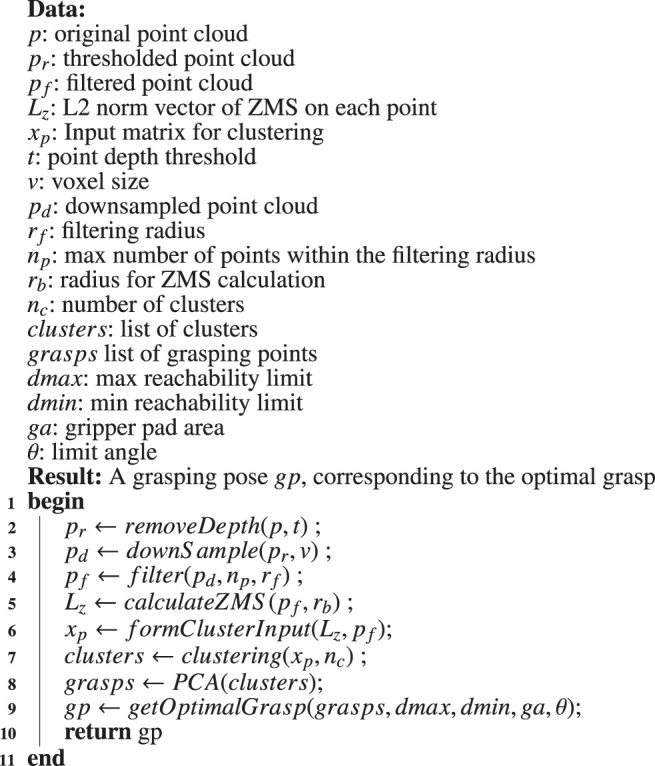

Algorithm 1: An outline of the steps used in the grasping algorithm (Mavrakis and Gao, 2019).

As outlined in our AADL model (Figure 1), there are two distinct software components that correspond to Algorithm 1. These are the imagepreprocessing and findoptimalgrasp components.

#### 4.1.1 Pre-Processing the Image

The algorithm is based on a point-cloud representation of a nozzle. Such a point cloud can be extracted by a depth sensor, stereo camera, or LIDAR sensor. It is assumed that the sensor faces the point cloud so as to make the nozzle stick out of the background body of the target. First, the nozzle cloud is segmented from the background by applying depth thresholding to the point cloud (line 2 of Algorithm 1). Every point with depth greater than the threshold value *d* is omitted, and the remaining cloud corresponds only to the target’s nozzle.

The cloud then gets downsampled to enable faster processing (line 3 of Algorithm 1). Square voxel downsampling is used, with voxel size of *v*. The resulting point cloud retains the overall geometric structure but has a reduced number of points. Finally, the downsampled cloud is then filtered to eliminate noise and isolated points (line 4 of Algorithm 1). Each point is checked against a ball neighbourhood of radius *r*_*f*_. If the neighbourhood of the point contains less than *n*_*b*_ points, then the point is discarded.

#### 4.1.2 Finding the Optimal Grasp

After the point cloud has been processed, the algorithm generates grasping points based on the nozzle’s surface characteristics. For each point, the *Zero Moment Shift* (ZMS) is calculated (line 5 of Algorithm 1). The ZMS is the distance 3D vector of a point from the mean within a ball neighbourhood of radius *r*_*b*_, and its norm represents a measure of surface smoothness. After the ZMS is calculated, we create a 4D vector for each point that consists of the 3D coordinate and the ZMS norm scaled by a constant *s*. The 4D vectors are used as input to a clustering algorithm, and so the nozzle surface is divided into graspable patches according to smoothness and vicinity (lines 6 and 7 of Algorithm 1). We apply *Principal Component Analysis* to each patch (line 8 of Algorithm 1), resulting in a 3D coordinate frame that corresponds to a robotic grasp, i.e. a pose for the robotic end-effector. The final grasp is selected from the grasp set according to reachability criteria (line 9 of Algorithm 1).

### 4.2 Encoding the Basic Data Types

**FIGURE 5:**
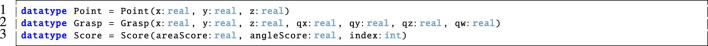
The basic datatypes for storing points, grasps and scores in our Dafny model.

We encoded our basic types using tuples in Dafny as shown in Figure 5. In particular, a Point is a tuple with three real number elements (line 1). A Grasp (line 2) has seven real number elements. Finally, a Score (line 3) has three real number elements. The first is an area score (this will be 1 if the calculated area is above the area threshold), the second is an angle score which is the score for the cluster and finally the index stores the index in the sequence of calculated grasps which corresponds to this score.

### 4.3 Image Pre-Processing

Our Dafny model specifically focuses on the imagepreprocessing and findoptimalgrasp components that are illustrated in the AADL model in Figure 1. Since we focus on these components, our verification task shall demonstrate that requirements R1.4, R1.5 (including R1.5.1 and R1.5.2) and R1.6 are met by our model of these software components. To ensure accuracy, we also encode assumptions relating to other requirements as necessary, particularly R1.3.2 as will be discussed later.

**FIGURE 6:**
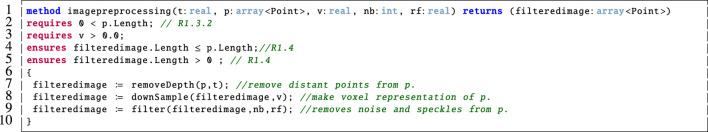
Dafny model of the imagepreprocessing component shown in Figure 1. This was originally part of a larger Dafny model in (Farrell et al., 2020) but it has since been refactored to conform to the system architecture described by the AADL model shown in Figure 1.

We begin by describing the imagepreprocessing phase. In our Python implementation, this involves employing three library functions for removing depth, down sampling and filtering the point cloud which is received from the camera. These calls are illustrated on lines seven to nine of Figure 6. We use the requires clause on line 2 to capture the assumption for this component that the input point cloud, p, shall not be empty, corresponding to R1.3.2 from Table 1. Here, we implement a point cloud as a Point array and so we specify that 0<p.Length . This directly corresponds to the FRETISH for this requirement as shown in Table 1. We also require that the value of the voxel size, v, be positive. Note that this was not listed as an official requirement in Table 1, but that it is necessary in order to verify the correct functionality of the system at implementation-level.

R1.4 is the only requirement that is specific to the imagepreprocessing component of the system and this is verified using the ensures clauses on lines 4 and 5 of Figure 6. In particular, we verify that the filtered image is non-empty and is no larger than the unfiltered point cloud, p. This is echoed in our Dafny encoding of each of these helper functions.

**FIGURE 7:**
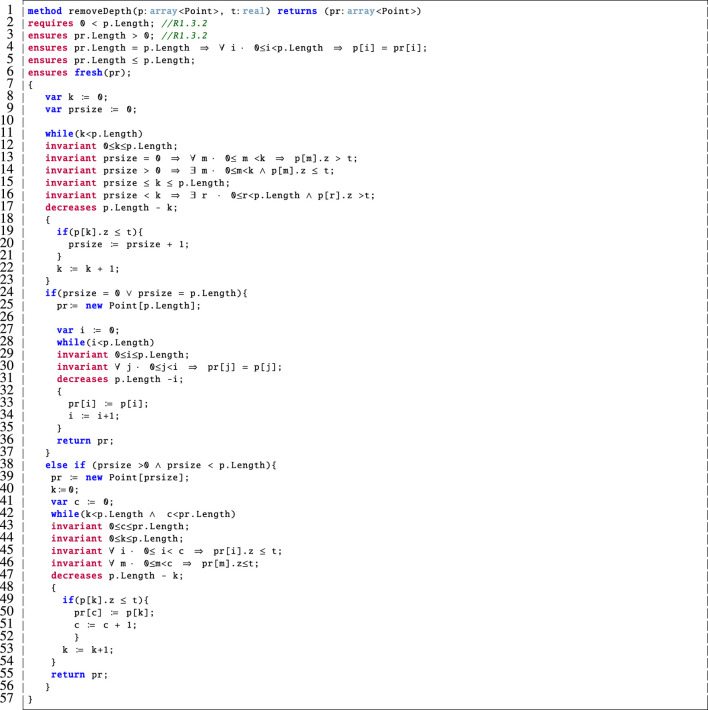
Dafny encoding of the removeDepth method.

Crucially, Dafny was not equipped with these library functions that were used in our Python implementation. Therefore, we provided simplified encodings of these in our Dafny model. As an example, we include the corresponding Dafny method for removeDepth in Figure 7.

This method takes a point cloud, p, and a real number threshold, t, as input. It then outputs a point cloud, pr, which is the filtered version of the input point cloud with all points whose z-value is above the threshold removed. We verified the correctness of this method and specified that it behaves correctly using the post-conditions on lines three to six and, via the associated loop invariants on lines 12–16, 29–30 and 43–46. In Dafny, loop invariants must hold before and after each loop iteration, and they are used by the prover to support the verification of the post-condition(s). We note the addition of the if statement on line 24 to our prior work (Farrell et al., 2020) to prove that the point cloud is never empty. In this case, if the removeDepth method would result in an empty point cloud then we return a copy of the input point cloud in its place. This ensures that R1.3.2 is not violated. A detailed description of this method follows:

Lines 1–6: This method takes as input a point cloud, p, and a real number threshold, t. It then outputs a point cloud, pr, which is a filtered version of the input point cloud with all points whose z-value is above the input threshold removed. The pre-condition on line 2 requires that a non-empty point cloud be input, this corresponds to R1.3.2 from Table 1. This is supported by the post-condition on line 3 which ensures that the output point cloud also be non-empty. Further, the post-condition on line 4 specifies that if the input and output point clouds are the same size then they are equal and (line 5) that the output point cloud is at most as large as the one that was input. Finally, our use of fresh in the post-condition on line 6 ensures that all of the elements of pr were all freshly allocated in the current method invocation. This was needed to verify the properties in imagepreprocessing because the outputs of one filtering step are input to the next. The inclusion of fresh is a modification of our original Dafny model from (Farrell et al., 2020) due to its more accurate encoding.

Lines 8–10: Here we declare and initialise a loop variable, k, and a variable to correspond to the size of the output array, prsize.

Lines 11–23: We cannot create an array of unspecified size in Dafny and, since we do not know the final size of the output array, pr, until we examine the elements of p, this loop counts up the number of elements in p that meet the condition to be included in pr. Namely, that their z-value is below the threshold, t, so they should not be filtered out. The invariant on line 13 specifies that if prsize is equal to 0 then none of the elements considered so far meet the criteria. On line 14, if prsize is greater than 0 then there are some elements that meet this criteria. We also verify that prsize remains within the allowed bounds on line 15. On line 16, we check that if prsize is less than the number of elements considered so far then there are some elements in p that do not meet the desired criteria. These invariants are used to verify that the loop functions correctly.

Lines 24–37: If the value of prsize is either equal to 0 or the length of p then we initialise pr to be the same size as p (line 25). Then the while loop on lines 28–35 makes pr an exact copy of p. This is verified by the invariant on line 30 which supports the post-condition on line 4. It is common practice in Dafny to have an invariant (e.g., line 30) which is a rephrasing of a related post-condition (e.g., line 4) in terms of a loop variable.

Lines 38–57: Otherwise, if prsize is a value other than 0 or is less than p.Length then we initialise pr to be of size prsize on line 39. The while loop on lines 42–54 then populates pr with those elements of p whose z-value is below the threshold, t. Again, there are associated loop invariants here that are used to prove that the loop functions correctly.

### 4.4 Find Optimal Grasp

**FIGURE 8:**
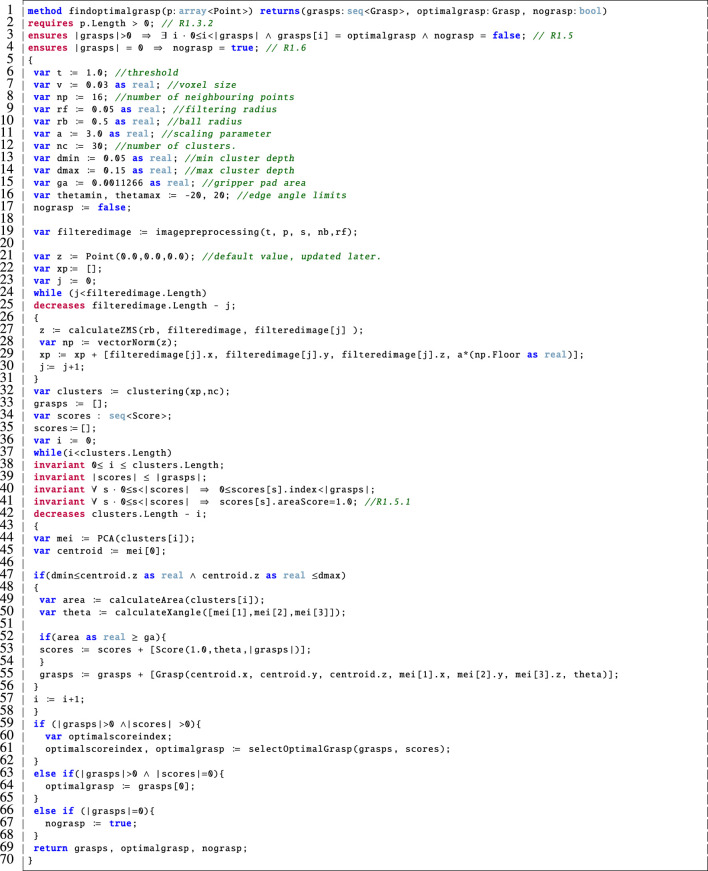
Dafny encoding of the findoptimalgrasp method.

The method presented in Figure 8 accounts for the main functionality of the findoptimalgrasp component shown in Figure 1. This corresponds to Algorithm 1 as outlined earlier. In particular:

Line 1: This method takes a point cloud, p, as input, it outputs the generated sequence of grasps, the optimalgrasp and a boolean flag which is used to alert the user if no suitable grasp was calculated.

Lines 2–4: The pre-condition on line 2 specifies that the input point cloud must not be empty, in agreement with R1.3.2. The post-condition on line 3 ensures that the optimal grasp is indeed a grasp that was calculated in accordance with R1.5. Then the post-condition on line 4 specifies that if there are no valid grasps returned then the nograsp boolean is set to true, as stipulated by R1.6. We note that in our previous version, a modifies clause was included here but, after more detailed analysis, we discovered that this was not necessary here since we no longer directly copy or modify p due to the inclusion of the imagepreprocessing component in Figure 1. This is a specific difference between this Dafny model and our original that was presented in (Farrell et al., 2020).

Lines 6–23: These variables correspond to those that were outlined in Algorithm 1. We have tried to maintain the nomenclature where possible but for implementation purposes we had to include additional variables. We decomposed *θ* as used in Algorithm 1 into thetamin and thetamax for implementation purposes on line 16 of Figure 8.

Lines 24–31: This while loop captures the functionality of lines five to seven of Algorithm 1 by calculating the ZMS, forming the correct input to the clustering method and then executing clustering with the appropriate inputs. Note that the decreases clause on line 25 is used to prove loop termination^2^ which is a basic program correctness property that Dafny requires us to preserve.

Lines 32–44: This part of the method calculates the potential grasps and assigns a score for each one using the criteria devised in (Mavrakis and Gao, 2019). We capture line 8 of Algorithm 1, which invokes PCA, here on line 44. The loop invariants shown here on lines 38–41 ensure that the loop variable remains within appropriate bounds (line 38), the correct number of scores are placed into the sequence of scores (line 39), that each score is matched to the corresponding grasp (line 40), and that only scores with an appropriate area component are stored (line 41), partially fulfilling R1.5.1 (offset of 1 cm). As above, line 42 is used to prove loop termination.

Lines 45–70: This part of the method corresponds to the function called on line 9 of Algorithm 1. In particular, lines 45–58 implement the score calculation. Once the scores and grasps have been calculated, we then call the selectOptimalGrasp method which chooses the optimal grasp based on the criteria in (Mavrakis and Gao, 2019) (lines 59–62). In case no grasp meets the ideal score, we simply return the first one in the sequence (lines 63–65) and if no grasps could be calculated then we toggle the nograsp boolean flag (lines 66–68).

**FIGURE 9:**
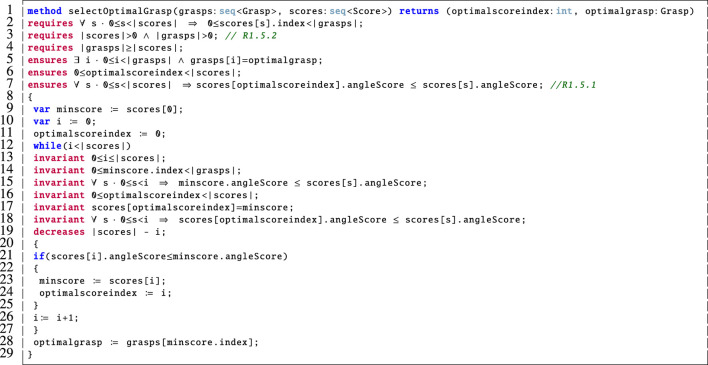
Dafny encoding of the selectOptimalGrasp method.

The findoptimalgrasp method above calls the selectOptimalGrasp method, illustrated in Figure 9, which models how the optimal grasp is selected from those that were computed based on the calculated scores. We describe its functionality as follows:

Line 1: Since it is important to ensure that the chosen grasp is optimal, this method takes the sequence of computed grasps and the associated scores as input and outputs the index of the optimal score (optimalscoreindex) along with the corresponding optimalgrasp.

Lines 2–4: These are the pre-conditions for this method. The first pre-condition (line 2) states that every index in the sequence of scores must be a valid index in the sequence of grasps. This is necessary so that we do not allow for the scores sequence to refer to a grasp that is not present in the sequence of grasps. The pre-conditions on lines 3 and 4 require that neither of the inputs are empty sequences and that there are at least as many scores as there are grasps in the input. Since this method is called by the findoptimalgrasp method (Figure 8), we must verify that these pre-conditions are met. This is achieved via the loop invariants on lines 38–41 of Figure 8 in support of R1.5.2.

Lines 5–7: These post-conditions ensure that the selected optimal grasp is indeed present in the input sequence of grasps (line 5) and that the optimalgrasp is indeed optimal because it is the one with the smallest angle score (line 7). Thus, we verify that R1.5.1 is met. We also ensure that the corresponding index is a valid index in the scores sequence (line 6).

Lines 9–11: Declare and initialise the necessary variables. We use minscore to keep track of the best score found so far.

Lines 12–29: The while loop on lines 19–28 is used to step through the scores sequence and keep track of the best score so far. Then we return the optimalgrasp as the one in the sequence of grasps at the identified index (line 28). The invariants on lines 13–18 ensure that the loop behaves correctly and are used by the prover to verify the earlier post-conditions (on lines 5–7). The loop variant on line 19 is used to prove that the loop terminates.

### 4.5 Verification Results and Discussion

Overall we were able to discharge all proof obligations automatically using version 2.3 of Dafny in version 1.48 of Visual Studio Code on Ubuntu 18.04.

A large variety of methods are typically used for verification in robotics (Luckcuck et al., 2019). In our case, we selected the use of the Dafny language, as its similarity to general coding paradigms and its syntax enable easier translation of the actual code used in the grasping algorithm to a Dafny program (Leino, 2017). Crucially, Dafny’s specification constructs and executable code are both written using the same syntax making it easy to communicate to non-expert users as remarked in our previous work (Farrell et al., 2020).

We found that Dafny was sufficiently expressive for the requirements that we needed to verify, although there are likely other tools or approaches that could have also been applied to this problem. Defining appropriate invariants can be difficult and often requires the skill of an experienced Dafny user. Approaches to automatically generate invariants is an active area of research and out of the scope of this paper. Such approaches include techniques that use abstract interpretation, which is actually used by the Boogie intermediate verification language that Dafny programs are automatically translated to for proof support (Barnett et al., 2004).

We used real numbers in our Dafny model which are supported natively. Interestingly, real numbers in Dafny are not treated as floating point numbers. They are the mathematical reals which are reasoned about using Z3 (Ford and Leino, 2017).

We note that, the Dafny tool support available in Visual Studio Code (Krucker and Schaden, 2017) and on the rise4fun website^3^ was useful, although the error messages could sometimes be unclear.

## 5 Runtime Verification

Runtime Verification (RV) is a lightweight formal verification technique which consists of checking the behaviour of a system while it is running (Leucker and Schallhart, 2009). With respect to other formal verification techniques, such as model checking (Clarke et al., 2001), RV has the advantage of being performant, and of not suffering from the state space explosion problem (typical in static verification). These two aspects make RV a suitable candidate in robotic applications where the system may have limited resources and can be too complex to be fully abstracted. Abstraction is usually required in static verification, as evidenced by our use of Dafny in the previous section which focused on two of the components in our AADL model in Figure 1, namely the imagepreprocessing and findoptimalgrasp components.

From a theoretical perspective, RV addresses the word inclusion problem (NLOGSPACE-complete for non-deterministic automaton (Hopcroft and Ullman, 1979)), where the objective is to identify whether a given trace of events belongs to the set of traces denoted by a given formal property (commonly referred to as the language of the property). The resulting verification process can be obtained in polynomial time considering the length of the trace to be analysed. This differs enormously from the problem tackled by other verification techniques such as model checking, where the objective is to exhaustively check whether the system under analysis satisfies (resp. violates) a given formal property. For example, model checking needs to analyse each possible system execution and to address the resulting language inclusion problem (PSPACE-complete for non-deterministic finite-automata (Sistla and Clarke, 1985)), by identifying whether the set of all possible traces which can be generated by the system execution is included in the set of traces denoted by a given formal property.

One of the most common ways to implement RV is through the use of runtime monitors. A monitor is a software component which can be automatically synthesised from a formal property, usually a temporal property which might be expressed using Linear-time Temporal Logic (LTL) (Pnueli, 1977). For example, (Bauer et al., 2006) presents an algorithm to synthesise a monitor as a Moore machine from an LTL property. The monitor’s job is dual: it gathers information from the system execution (the trace), and it checks such a trace to conclude whether the system execution satisfies (resp. violates) the property under analysis.

Given a trace of events and a property to verify, a monitor returns 1) *⊤*, if the trace has enough information to conclude that the system satisfies the property, 2) ⊥, if the trace has enough information to conclude that the system violates the property, 3) ? otherwise. Depending on the formalism used to denote the properties, the third outcome can be split into: (iiia) ?_*⊤*_, if the trace has not enough information to conclude neither that the system satisfies nor violates the property, but the current trace is currently satisfying the property, (iiib) ?_⊥_, if the trace has not enough information to conclude neither that the system satisfies nor violates the property, but the current trace violates the property.

Consider the following example. If we have the Past LTL property ■ *a* (*past always*), which means “*a* was always true in the past”. Given the trace *aaa*, the monitor returns ?_*⊤*_, since currently the trace is satisfying the property by having always *a* true in the past, but we do not have enough information to conclude *⊤* yet, because there is no guarantee that the system will continue to satisfy the property. Indeed, a possible continuation of the trace could be *aaab*, which would violate the property and ao the monitor would return ⊥. Considering another example, given the Past LTL property ♦ *a* (*past eventually*), which means “*a* was once true in the past”. Given the trace *bb*, the monitor would return ?_⊥_, since currently the trace does not satisfy the property, in fact *a* was never true in the past. But, if we continue to analyse the system, we might find a continuation such as *bba* that satisfies this property and the monitor would then return *⊤*. These two simple examples demonstrate that *⊤* and ⊥ are final verdicts (trace continuations do not change the outcome), while ?_*⊤*_ and ?_⊥_ are temporary verdicts that can change depending on how the trace evolves.

### 5.1 ROSMonitoring

RV is a useful candidate for verifying formal properties in robotic scenarios. The *de facto* standard for deploying robotic applications is the Robot Operating System (ROS) (Quigley et al., 2009). ROS provides libraries and tools to help software developers to create robotic applications. It provides hardware abstraction, device drivers, libraries, visualisers, message-passing, package management, etc.^4^. ROSMonitoring is a framework for performing RV of ROS applications. ROSMonitoring allows the user to add monitors to ROS applications. These monitors intercept the messages that are exchanged between components, called “ROS nodes”^5^, and check whether the relevant messages conform to a given formal property.

**FIGURE 10:**
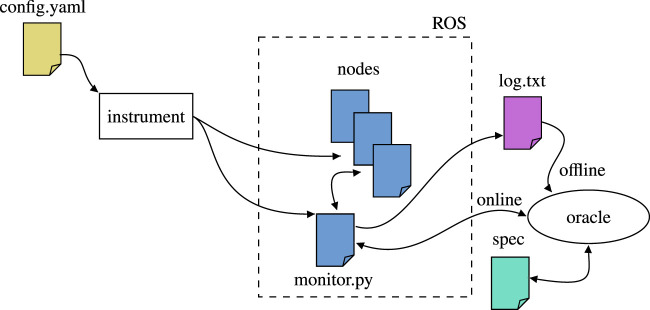
High-level overview of ROSMonitoring (Ferrando et al., 2020). Everything starts with a configuration file that guides the instrumentation process, in which the ROS nodes are modified (if necessary) and the monitor code is synthesised. Then, depending on the user’s choice, RV is carried out offline, where a log file is generated for future analysis, or online, where the intercepted ROS messages are propagated to an oracle component for incremental analysis.

Figure 10 describes the ROSMonitoring pipeline. ROSMonitoring automatically generates monitors following an input configuration file. These monitors intercept messages and report to the external oracle (the entity that has access to the formal property to verify). In the following we describe these three different aspects more in detail.

#### 5.1.1 Instrumentation

ROSMonitoring starts with a YAML configuration file to guide the instrumentation process required to generate the monitors. Within this file, the user can specify the communication channels, called “ROS topics”, that are to be intercepted by each monitor. In particular, the user indicates the name of the topic, the ROS message type that is expected in that topic, and the type of action that the monitor should perform. After preferences have been configured in *config. yaml*, the last step is to run the generator script to automatically generate the monitors and instrument the required ROS launch files.

#### 5.1.2 Oracle

ROSMonitoring decouples the message interception (monitor) and the formal verification aspects (oracle) and it is highly customisable. Different formalisms can be used to represent the properties to verify, including Past MTL, Past STL, and Past LTL (MTL, STL, and LTL with past operators respectively); the latter is one of the formalisms used by FRET to formally denote requirements (as shown on the right hand side of Figure 2). Given a requirement in FRET, we generate a Past LTL property and synthesise the corresponding monitor in ROSMonitoring to perform RV. Using the formalism of choice, an external entity can be created to handle the trace of events reported by the monitors in ROS (generated through instrumentation). ROSMonitoring requires very few constraints for adding a new oracle. It uses JSON as a data-interchange format for serialising the messages that are observed by the ROS monitor. Thus, an oracle will parse the JSON messages, check whether they satisfy or violate the formal property, and report back to the ROS monitor.

#### 5.1.3 ROS Monitor

The instrumentation process generates monitors to intercept the messages of interest. Each monitor is automatically generated as a ROS node in Python, which is a native language supported in ROS. If the monitor finds a violation of the property under analysis, it publishes a warning message containing as much information as possible about the violated property. This warning message can be used by the system to handle the violation and to react appropriately.

In our experiments, we focused on offline RV. Rather than checking the messages while the system is being executed, the monitors parse log files containing the messages that were generated during executions. The verification process does not change, from the viewpoint of a monitor to check a trace online at runtime or offline using log files is exactly the same. In this paper, we preferred to follow the offline approach in order to focus on the formal aspects of the properties and the corresponding requirements. The extension to be applied online is simple and straightforward to achieve; we left it out of this work to improve clarity, since we are not considering failure handling at this stage. In the future, we intend to apply online monitoring, and to modify the system to be aware of the monitors’ outcome, in order to properly react if requirements are violated.

### 5.2 Monitoring of Requirements

**FIGURE 11:**
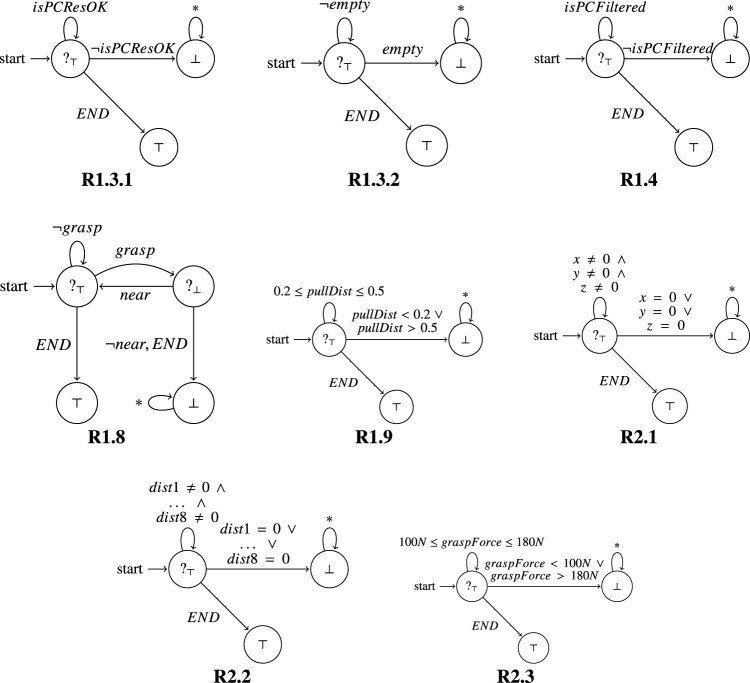
Monitors synthesised from the requirements listed in Table 1 are represented as automaton.

Requirements 1.3.1, 1.3.2, 1.4, 1.8, 1.9, 2.1, 2.2, 2.3 in Table 1 have been verified through RV. The resulting monitors are reported in Figure 11. In the following we describe some of them more in detail.

Requirement 1.3.2: The point cloud shall not be empty.

Past LTL: ■ *¬empty*.

In Figure 11, for this property (R1.3.2), the monitor starts in a state where as long as it observes *¬empty* the property is considered currently satisfied (?_*⊤*_). Indeed, as long as the point cloud is not empty, the system behaves correctly. If *empty* is observed, denoting that the point cloud is empty, we have a violation of the Past LTL property, and the monitor moves to the state with output (⊥). In this state the monitor can observe any kind of event (*) without changing its output. We also have a special event (*END*) representing the end of execution, meaning that the system has been correctly terminated. When the system ends, a current verdict becomes final (since there are no further continuations); in this case ?_*⊤*_ becomes *⊤*.

Requirement 1.8: The SVA shall capture the TGT at the BGP.

Past LTL: ■ (*grasp* ⇒ *near*)

In Figure 11, for this property (R1.8), the monitor starts in a state where as long as it observes *¬grasp* the property is considered currently satisfied (?_*⊤*_). Indeed, as long as the gripper does not grasp anything, there is nothing to analyse for the property (it is trivially satisfied). Then, when *grasp* is observed, the monitor moves to the state with output (?_⊥_). The reason is that we are currently in a situation where we know that the gripper has grasped the target (TGT), and to verify that it was right to do so, the monitor must also observe that the gripper is near the BGP. If it is (*near* is observed), then the monitor goes back to the initial state; otherwise (*¬near* or *END* are observed), the monitor moves to the state with output ⊥. In fact, if the gripper is not close to the BGP then the system is violating the property. As in the previous requirement, when *END* is observed, meaning that the system has been correctly terminated, the current verdicts become final verdicts. In this case if the monitor is in the initial state where it is currently satisfying the property, then the monitor moves to the state with output *⊤*. Instead, if the monitor is in the top right state when the system ends, then the monitor moves to the state with output ⊥, since the system was not currently satisfying the property. These monitors were deployed and we describe our experimental results in the next section. In particular, the monitors helped us to uncover some issues with the requirements and we discuss these in Section 7.5.

## 6 Experimental Results

To test our verification architecture, we set up a debris capturing scenario, and verify the capturing process both in a simulator and on a real (physical) orbital robotics testbed. The capturing scenario includes a chasing spacecraft (service vehicle SV) equipped with a 6-DOF robotic manipulator, a 2-fingered gripper and a depth sensor for point cloud extraction. The captured target is an AKM, modeled after the Thiokol STAR 13b family of rocket stages (Orbital ATK, 2016). The target was selected because of its low mass and simplistic shape, that enables easier modelling in simulation and emulation on the testbed. It also serves as a realistic case for debris removal, as similar AKMs from past launches remain in orbit, sometimes even decades later. In all cases, the SV arm is tasked with generating a grasping point on the target nozzle, moving the gripper towards the grasping point, executing the grasping, and pulling the target back for a specified distance. During the entire process, we use the monitors that were presented in Figure 11 to ensure that the system meets the requirements listed in Table 1.

### 6.1 Including the Runtime Monitors

The monitors that were presented in Figure 11 have been implemented using ROSMonitoring and applied to the simulation and real (physical) testbed. Section 5 focused on the theoretical aspects of a monitor. Here, we describe some of the more practical aspects. Specifically, we now consider the structure of the messages that are observed by a monitor. In the following, we provide an example of a trace of events observed in the verification of requirement R1.9.

**{“pullingDistance”: 0.3011664608030819814, “t”: 177.44376802444458}**

Each trace contains a JSON^6^ event. We used JSON because it is the standard data exchange format used in ROSMonitoring to propagate messages from ROS to the oracle. A ROS message can be automatically translated into JSON format using a custom Python library^7^. In this example, the log file contains the events which report the current pulling distance of the robotic arm (∼0.3 cm) and the time at which each event has been observed^8^ (∼177 s). As shown, the events in the trace satisfy R1.9, indeed the pulling distance is always within the desired range.

Considering the previous trace, the corresponding output of the monitor is:

**The event \{“pullingDistance”: 0.3011664608030819814, “t”: 177.44376802444458\} is consistent.**

Current outcome:?_*⊤*_

Here, we have the monitor’s current outcome for each observed event. If we look at the monitor representation in Figure 11, it is easy to see that by consuming these events we remain in the initial state, where the outcome is ?_*⊤*_. Meaning that the trace currently satisfies the requirement. Similar results were observed for all of the monitors depicted in Figure 11.

### 6.2 Simulation Verification

**FIGURE 12:**
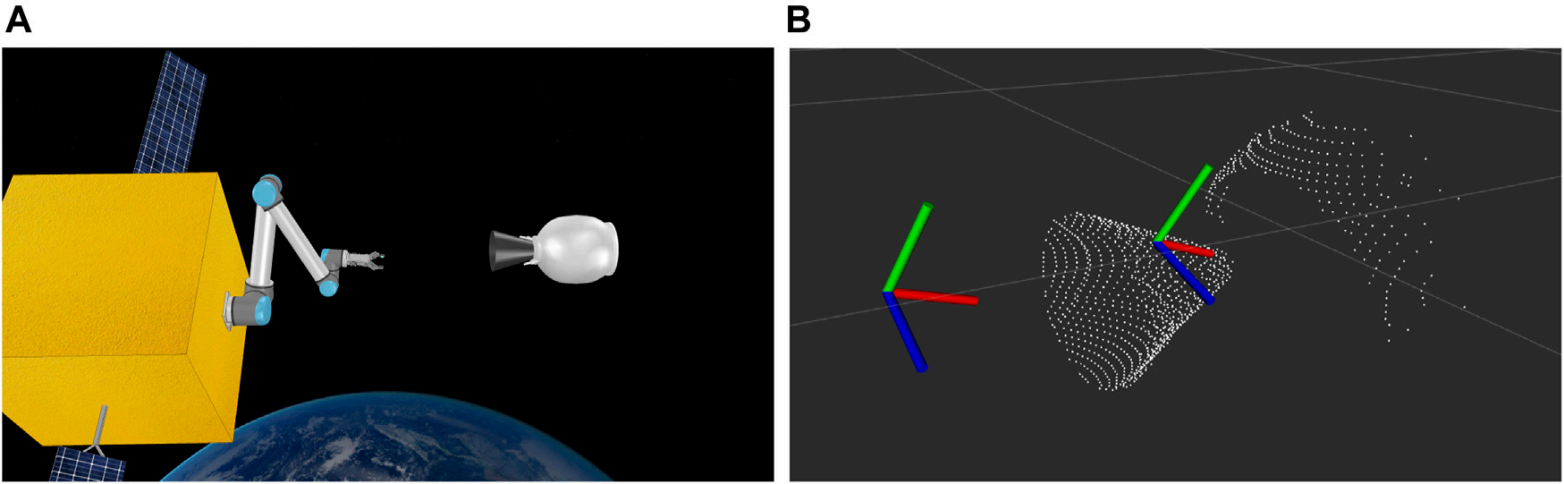
Simulation setup **(A)** and detected grasp and approaching pose on the nozzle point cloud **(B)**.

Simulations continue to play an important role in verifying and validating robotic systems (Luckcuck et al., 2019). As such, we use the V-REP simulator (Rohmer et al., 2013) to set up the simulated capturing scenario, as shown in Figure 12. The scene is weightless, and we use the Newton physics engine, available in V-REP to mimic the space environment. The SV has a mass of 500 kg, corresponding to a small to medium sized spacecraft, and the target has a dry mass of 9 kg, in accordance with the technical specifications provided in the manufacturer’s catalogue.

For simplicity, we assume that the target (TGT) and the chasing spacecraft (SV) have matched their attitude and rotational velocity, i.e. the TGT appears completely motionless as seen from the camera of the SV. In reality, this can be achieved by orbital maneuvering of the SV using its thrusters.

**FIGURE 13:**
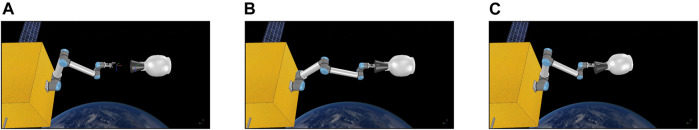
Reaching the approaching pose **(A)**, grasping pose **(B)** and final pulling pose **(C)**.

Figure 12 shows the detected point cloud of the target, as well as the grasping pose generated after using the algorithm of Mavrakis and Gao (2019). The robot is tasked with reaching the grasping pose through an approaching pose of the same orientation, but with an offset of 0.3 m towards the SV body. The approaching pose is also shown in Figure 12. The grasping process is illustrated in Figure 13. The robot begins from an initial pose, and ensures that requirements R1.3 (including R1.3.1 and R1.3.2) and R1.4 are met by examining the point cloud resolution and data size. R1.5 and R1.6 are verified formally using Dafny on the grasping algorithm pseudocode.

The SV then reaches the approaching point and the grasping point. Throughout the motion, the distance between each of the SV joints and the grasping point is monitored, to ensure that there is no collision between the SV body and the TGT (R2.1 and R.2). The SV then closes its gripper to capture the TGT. By checking the distance of the gripper tip and the grasping point, the SV ensures that R1.8 is preserved. The grasping force is monitored by simulated force sensors placed on the fingertips of the gripper, making sure that R2.3 is verified. After closing the fingers, the SV stores the gripper position, and starts pulling the target towards the satellite body. After pulling, the SV checks the new gripper position and compares it with the saved one at the start of the pull, to verify R2.3. The requirements were met for the whole capturing duration.

### 6.3 Testbed Verification

To verify the capturing scenario using real robots, we utilised the STAR-Lab Orbital Robotics Testbed (Hao et al., 2019). The testbed consists of two UR5 robots, the SV and TGT arm. The SV arm is mounted on top of a 2 m long track that enables motion in a 2D plane, emulating an approaching spacecraft. The TGT arm can emulate the orbital dynamics of a target mounted on its end-effector, and reproduce the motion of a weightless target when a force is applied to it. The SV arm has a Robotiq 2F-85 gripper for capturing, and an Intel Realsense D435 depth sensor mounted over its end-effector for point cloud capturing.

**FIGURE 14:**
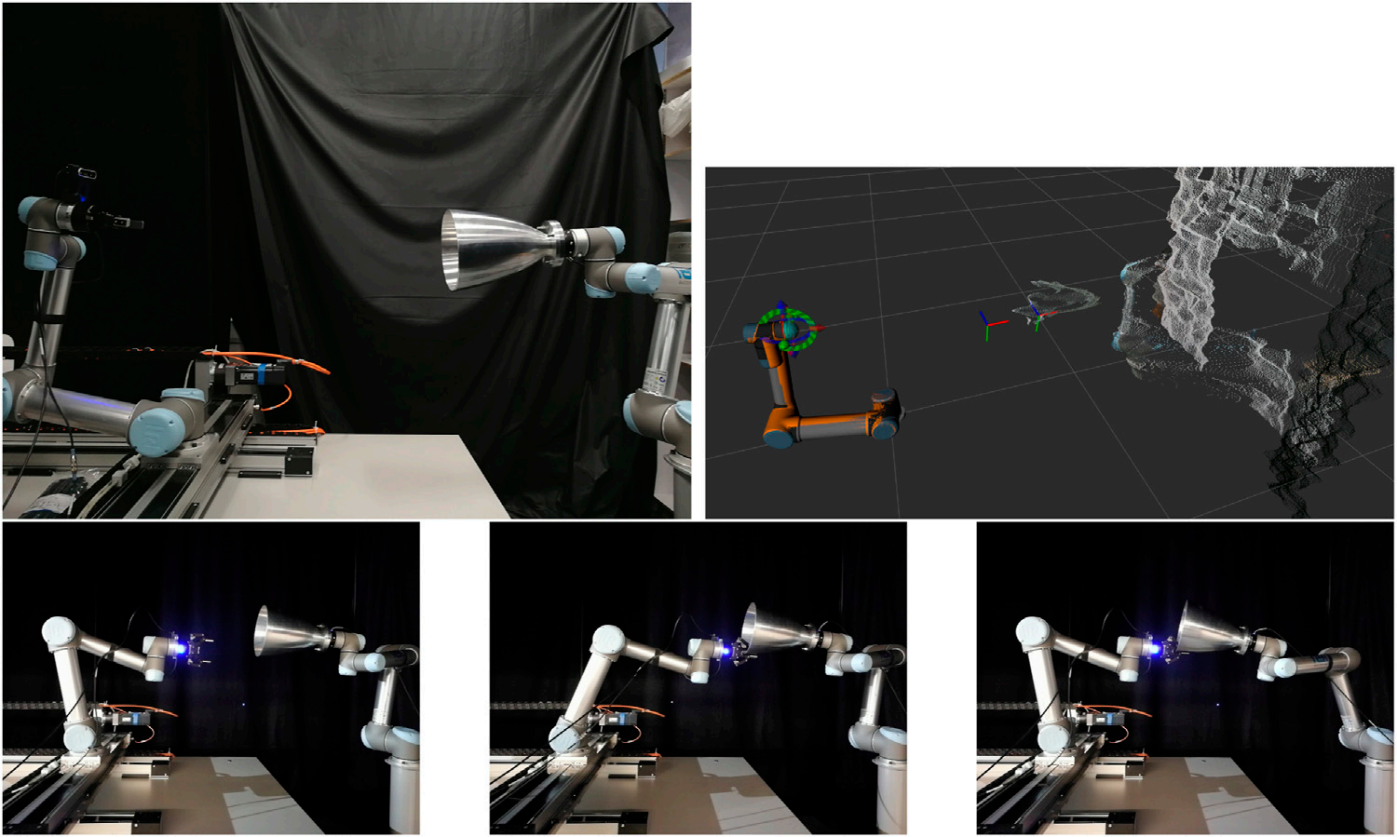
Testbed setup (top left) and detected grasp and approaching pose on the nozzle point cloud (top right). Approaching pose (bottom left), grasping pose (bottom middle) and final pulling pose (bottom right).

The grasping process is the same as the simulation, with the robotic arm going through the approaching, grasping and pulling poses sequentially. The starting point, detected point cloud, approaching pose and grasping pose are shown in Figure 14. The capturing and pulling motion is also shown in Figure 14. The requirements were checked for the physical in the same way as for the simulation. A difference lies in the grasping force measurement, where it is provided by Robotiq in the programming SDK of the gripper, by measuring the gripper motor current. In the end, the requirements were met for the whole grasping process.

### 6.4 Fault Injection

We conducted a capturing experiment where we intentionally injected a fault into the system to demonstrate the effectiveness of the monitors in identifying cases where the requirements are violated. Specifically, we reduced the grasping force used by the gripper to grasp the target. The gripper captured the target with a grasping force of 40 N, substantially less than the lower limit of R2.3. As a result, the applied force was not able to hold the target because it slipped through the gripper fingers during the pulling phase, and the SV lost contact with the TGT. This fault was correctly identified by the monitors for the requirements R1.9 and R2.3, approximately 134 s after the initial detection of the grasp.

We note how the property is initially satisfied (grasping force is within range and the target is grasped), but then, at approximately 134 s the grasping force observed by the monitor is ∼40N, which is outside of the range allowed by R2.3. We observe how this causes the gripper to lose contact with the target, indeed right after observing the wrong grasping force, the monitor observes the event with “isTargetGrasped” set to false. The monitor recognises these events, and reports them as inconsistent. The outcome becomes ⊥, as reported in Figure 11, where a violation sends the monitor to the state ⊥, where any event can be observed without changing the final verdict.

In this section, we discussed our experimental evaluation and use of the runtime monitors that were developed in Section 5 to verify our system, both in simulation and on the physical test bed.

## 7 Discussion

In this paper, we reported on our use of both non-formal (AADL) and formal (FRET, Dafny and ROSMonitoring) tools for the analysis of a previously developed autonomous debris grasping system. In this section, we describe the benefits/modifications that each approach offered, the gaps that were identified in the requirements and discuss our approach to post-implementation verification.

### 7.1 Architecture Analysis and Design Language

Firstly, we devised an AADL model of the system. This was primarily to act as a point of reference where we decomposed the system into its constituent parts (both hardware and software). Through this process, with a view to include runtime monitors for the system we refactored our original algorithm to split up the functionality of imagepreprocessing and findoptimalgrasp which were originally encoded as a single entity in our algorithm. This refactoring facilitated the use of runtime monitors and the definition of detailed requirements for each of these components. It was beneficial to use this AADL model when we were defining the system’s requirements because it allowed us to focus on specific components of the system when devising particular requirements. It also provided a reference point for variable names and component names to be used in natural language requirements.

### 7.2 Formal Requirements Elicitation Tool

We used FRET to formalise our requirements. Previously in (Farrell et al., 2020), we had identified three requirements specific to the software itself. However, in this paper, we adopted a much broader view of the system which allowed us to identify and formalise many more requirements as illustrated in Table 1. Our goal was to utilise formal verification techniques, specifically Dafny and ROSMonitoring, to verify our system so the intermediate FRET representation was desirable as it more closely resembled formal requirements than their natural-language description. Also, the semantics given by FRET could be used in the development of runtime monitors with ROSMonitoring.

### 7.3 Dafny

We had originally developed a Dafny model of the imagepreprocessing and findoptimalgrasp components. However, like the implementation, we refactored our Dafny model to match the architecture shown in Figure 1 with little consequence to the verification of the model. This refactoring forced us to include some further specification constructs for the helper functions that are used by the imagepreprocessing component but this was relatively straightforward. Interestingly, some of the specification constructs that were specified in the original Dafny model corresponded to the requirements identified in Table 1.

### 7.4 ROSMonitoring

We used the FRET requirements to synthesise a catalogue of runtime monitors, using ROSMonitoring, for a subset of the requirements shown in Table 1. Some of these requirements were also verified using Dafny (e.g. R1.3.2). However, most were focused on requirements that would not be easily verified in a static verification tool similar to Dafny. It is important to stress that the process of developing these monitors was simplified by our use of FRET since it provided a concise semantics for each of these requirements. We executed these monitors offline (using log files) both for the simulation and the real system to ensure that the requirements were preserved. We also note that, due to the novelty of the autonomous grasping system, it was not possible to provide detailed requirements for specific components without having an implementation.

### 7.5 Gaps in the Requirements

Interestingly, the development of some of these monitors allowed us to identify gaps in our requirements that were subsequently captured and formalised using FRET. Specifically, the requirements R1.9 and R2.3 were initially considered to be violated by the corresponding monitors when applied to the real testbed. This was mainly caused by hardware restrictions. In R1.9, the initial requirement stated that the pulling range should have been between 0.3 and 0.5 cm. Even though this was obtainable in simulation, it was not in the real system because the robotic arm did not allow it (safety mode enforced a shorter pulling range). This helped us to understand the hardware limitations, and it caused us to update the pulling range in this requirement. The range has been updated to 0.2–0.5 cm, which was derived from the observation of what happened in similar real case studies.

In R2.3, the grasping force was stated to be 180 N. Again, this requirement was met in simulation, but not in the testbed. As for R1.9, this violation was a result of hardware restrictions. Specifically, the gripper’s maximum force is 180N, and the hardware could not always reach it. This was a violation of R2.3, and it was correctly observed by the corresponding monitor. Following this observation, the requirement was modified to constrain the grasping force to the range 100–180 N. Such a range has been determined through experiments.

There were three requirements (R1.1, R1.2 and R1.7) listed in Table 1 that we did not formally verify or monitor. Specifically, R1.1 was verified by construction on the test bed where we physically placed the camera 0.5 m away from the TGT. With respect to R1.2, we didn’t impose an initial velocity on the TGT in either the simulation or the testbed so this requirement was met by design. However, if this system were to be deployed then we would have to encode a way of determining whether the TGT was indeed motionless and potentially synthesise a monitor for this. Finally, R1.7 was verified via physical testing and visual examination. We intend to investigate whether it would be possible to develop a runtime monitor for this property as future work.

### 7.6 Post-implementation Verification

The usual approach that is advocated is linear, where the system architecture is defined, requirements are elicited/formalised, formal models of system components are verified, monitors are generated and finally, the system is implemented. However, it is often the case that system verification is forgotten about until the development is almost finished. This tends to make the verification process more difficult, particularly as system complexity increases (Luckcuck et al., 2019). In our case, we were in the latter category. The system was almost complete when we sought to verify it.

Contrary to the usual difficulties caused by taking such an approach, we were able to reverse engineer our verification process. Notably, our system was not overly simple but it was not too complex in terms of structure and we were willing to make small changes to the implementation in order to streamline the verification phase. We conclude that, although it would have been beneficial to develop the system with verification in mind from the outset, it was also useful from a verification perspective to have an implementation to examine from the beginning. When defining the requirements, we were able to query the system to find special cases where the requirements were violated and to thus refine the requirements and associated formal models based on this. In a way, we used both the implementation artefacts and the verification artefacts to inform one another. From the perspective of scaling this approach to a more complex system, the main factor would be the degree of modularity in the system. From this work, it has become clear that the more modular a system is then the easier it is to analyse and verify.

## 8 Conclusions and Future Work

In this paper, we presented our approach to formally verifying a previously developed autonomous debris grasping system. We used AADL to devise a model of the system which was used as a basis for defining natural language requirements that were subsequently formalised in FRET. For the software components defining the autonomous behaviour, we developed and verified their functionality using the Dafny program verifier. We then generated a suite of runtime monitors using ROSMonitoring and used these in an offline fashion to examine log files from both the simulation of the system and the physical testbed. Further, to validate these monitors we injected a fault into the system which was successfully identified by the monitors as a violation of the defined requirements.

Our definition of requirements for this system followed a usual, safety-focused approach. However, cyber-security is increasingly becoming a concern for space systems and, as future work, we intend to analyse and verify requirements of this system related to security, taking inspiration from (Farrell et al., 2019a) and (Maple et al., 2020).

## Data Availability

The original contributions presented in the study are included in the article/supplementary material, further inquiries can be directed to the corresponding author.

## Footnotes

1 Available: https://github.com/autonomy-and-verification-uol/ROSMonitoring.

2 Each iteration of the loop decreases the value of filteredimage.Length-j until it reaches zero.

3
https://rise4fun.com/dafny/.

4
http://wiki.ros.org/.

5 ROS is node-based, each robot can be composed of multiple nodes. For example, each of the components in the AADL model in Figure 1 correspond to a node or a group of nodes in our ROS-based system.

6 JavaScript Object Notation (www.json.org).

7 Repository: https://github.com/uos/rospy_message_converter.

8 The trace has been previously generated and the monitor analyses the log files from past executions.
